# Food Safety in Post-COVID-19 Pandemic: Challenges and Countermeasures

**DOI:** 10.3390/bios11030071

**Published:** 2021-03-04

**Authors:** Weimin Zhang, Huiyu He, Lin Zhu, Guozhen Liu, Long Wu

**Affiliations:** 1 Key Laboratory of Food Nutrition and Functional Food of Hainan Province, College of Food Science and Engineering, Hainan University, Haikou 570228, China; zhwm1979@163.com; 2 College of Food Science and Technology, Huazhong Agricultural University, Wuhan 430070, China; cyp89@126.com (H.H.); zlin0715@126.com (L.Z.); 3 School of Life and Health Sciences, The Chinese University of Hong Kong, Shenzhen 518172, China; liuguozhen@cuhk.edu.cn; 4 Key Laboratory of Fermentation Engineering (Ministry of Education), National “111” Center for Cellular Regulation and Molecular Pharmaceutics, College of Bioengineering and Food, Hubei University of Technology, Wuhan 430068, China

**Keywords:** SARS-CoV-2 transmission, food supply chain, food samples pretreatment, analytical techniques, food control

## Abstract

Understanding food safety hazard risks is essential to avoid potential negative heath impacts in the food supply chain in a post-COVID-19 pandemic era. Development of strategies for virus direction in foods plays an important role in food safety and verification. Early warning, tracing, and detection should be implemented as an integrated system in order to mitigate thecoronavirus disease 2019 (COVID-19) outbreak, in which the detection of severe acute respiratory syndrome coronavirus-2 (SARS-CoV-2) is critical as it not only concerns screening of populations but also monitoring of possible contaminated sources such as the food supply chain. In this review, we point out the consequences in different aspects of our daily life in the post-COVID-19 pandemic from the perspective of the food supply chain and the food industry. We summarize the possible transmission routes of COVID-19 in the food supply chain before exploring the development of corresponding detection tools of SARS-CoV-2. Accordingly, we compare different detection methods for the virus in foods, including different pretreatments of food matrices in the virus detection. Finally, the future perspectives are proposed.

## 1. Introduction

Dating back to the outbreak of the coronavirus disease 2019 (COVID-19), a large number of unexplained pneumonia cases were first discovered in December 2019, and most of them had an exposure history to the South China Seafood Market. The non-recognized and undetected virus was able to induce human respiratory infections and severely endanger the lungs and other organs. Later, it was found that the virus belonged to a sub-branch of coronavirus, named severe acute respiratory syndrome coronavirus-2 (SARS-CoV-2) [1]. Since then, SARS-CoV-2 has raised widespread concern worldwide, and hundreds of studies have been conducted by researchers.

The preliminary study showed that COVID-19 is easily transmissible from person to person via cough, sneeze, respiration, or exhalation [2], which requires physical prevention tactics such as social distancing or wearing masks. Further, by studying its genome sequence, protein structure, and infection behavior, it was found that SARS-CoV-2 is similar to the SARS-CoV and Middle East respiratory syndrome virus (MERS-CoV) [3]. The main difference lies in the key mutations in the S protein receptor-binding domain on the virus surface, which greatly increases the binding force of SARS-CoV-2 and the cell surface ACE2, thus leading to COVID-19, a highly contagious disease [4].

Coronavirus is an enveloped RNA virus that has caused widespread infections in the past, including severe acute respiratory syndrome coronavirus (SARS-CoV) in 2003 and Middle East respiratory syndrome virus (MERS-CoV) in 2012 [5]. In fact, SARS-CoV-2 is the seventh member of the coronavirus family and the only one with high infections among human beings [6]. On 11 March 2020, the World Health Organization (WHO) listed COVID-19 as a global pandemic [7,8]. At present, the number of COVID-19-infected people is increasing sharply, while effective vaccines for COVID-19 are limited. Hence, constant attention is still required to reduce the risks of the virus on all walks of life, especially the food that is closely related to people.

Limited proof has been found that SARS-CoV-2 can be transmitted through food, but it does not mean that SARS-CoV-2 will not be transmittable during the food and food chain [9]. The “COVID-19 and Food Safety: Guidelines for Food Companies” jointly issued by the Food and Agriculture Organization of the United Nations and the World Health Organization on 7 April 2020 clarified that COVID-19 had brought tremendous impact and changes to the food industry due to the complex network system of food production, supply, and consumption [10]. Thus, the food industry should seek for insights to solve the hygiene and safety management problems, and it is essential to develop methods for detection of SARS-CoV-2 that will be helpful for the prevention and control of the COVID-19 pandemic.

**Figure 1:**
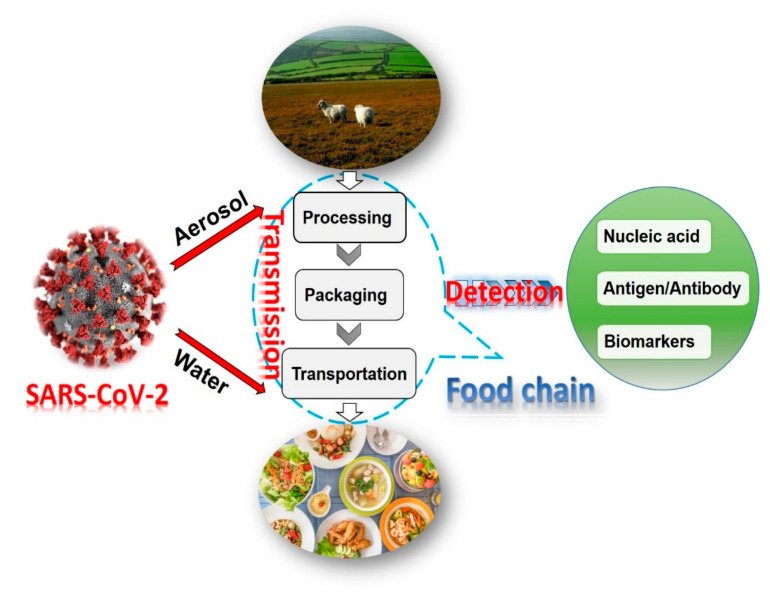
Schematics for the critical stages of the food supply chain from farm to table and methods for detection of severe acute respiratory syndrome coronavirus-2 (SARS-CoV-2) [9,10].

This review discusses the current status of food safety in the COVID-19 pandemic and reveals its possible transmission in the food and food chain. Then recent advances in development of analytical methods for detection of SARS-CoV-2 are summarized and discussed (Figure 1). Finally, perspectives on food safety in post-COVID-19 pandemic are proposed. This review provides a comprehensive guidance for people who would like to work on food safety from sample treatment to detection.

## 2. Transmission in Food Supply Chain

Three main transmission means of SARS-CoV-2 have been proposed and discussed, namely, human-to-human contact transmission [11,12], aerosol transmission [13,14], and droplet transmission [15]. Moreover, it has been suggested that the virus can transmit via the digestive tract [16], but its role and significance need further observation and research. Direct human-to-human contact, such as shaking hands with an infected person or touching objects contaminated by an infected person, can be a possible transmission route [17,18]. In addition, an infected person can infect other people with droplets when they sneeze or cough and may also expose virus-containing droplets to the air and form aerosols [19].

**Figure 2:**
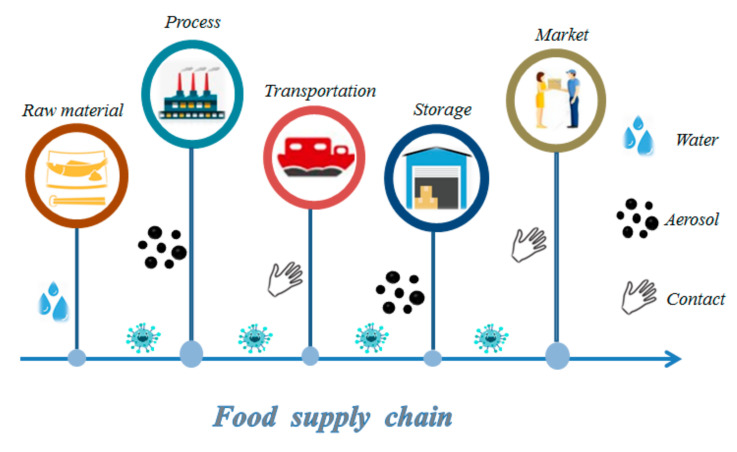
The possible transmission of SARS-CoV-2 in a food supply chain via three main means: water, aerosol, and contact.

In the previous outbreak of MERS and SARS-CoV viruses, food was considered less likely to be a route of transmission [20,21]. Although the coronavirus is mainly transmitted through droplets and close contact among humans, the possibility of transmission through water, bioaerosols, and food should not be ignored [22]. For example, an infected person can directly sneeze or cough coronavirus on the fresh food products or food packaging. It was reported that SARS-CoV-2 was detected on the surface of a salmon chopping board [23]. In this regard, potential spread of the virus is highly possible in the food supply chain (Figure 2).

Limited attention has been focused on the transmission of SARS-CoV-2 in food and the food supply chain [24]. For the virus sources, recent studies reported that wild animals (bats, snakes, pangolins, etc.) may be intermediate hosts that transmits the virus [25]. Even domestic animals such as dogs and cats are easily infected by SARS-CoV-2 [26,27]. In this regard, bad eating and living habits can be a risk for the virus spreading into the human population. In food processing, the infected employees may contaminate food and equipment, thus increasing the risk of spreading the virus to the food products and the whole factory [28,29]. Studies have found that samples collected from the air outlet fans and toilet sites (toilet bowl, sink, and door handle) have positive results, indicating that the virus-laden droplets may be deposited on the vent and other equipment [30]. When factories are in poor ventilation and dense environment, aerosols carrying the virus can be transmitted through the air to workers [31] or deposited on the container and packaging of the product [28]. The virus can survive long term on dry surfaces, sometimes up to several months, due to the difficulties in thoroughly cleaning up food packaging and containers [32]. Research has shown that SARS-CoV-2 is more stable on plastic and stainless steel than copper and cardboard, and the live virus can be detected within 72 h after being applied to these surfaces [33]. Thus, the risk of transmission from infected persons to food products is likely high, as evidenced by positive results in solid surfaces (floor, table, window).

Further, when the food is contaminated by the virus, the risk of infection may increase during the transportation due to the lack of strict control of the transportation pathway. In the early stages of the outbreak, most of the initial infected cases were due to the contact of the contaminated seafoods or animals in the South China Seafood Market [29]. As demonstrated, the virus may exist in food for a long time, which can survive for several days at 4 °C, and the infectivity can remain up to 2 years during frozen transportation at −20 °C [34,35]. Thus, the possibility of transmission through food chain is very high, especially the frozen food. On 3 July, the Dalian and Xiamen Customs in China claimed that the container surface of the frozen South American white shrimp manufactured by an Ecuadorian enterprise were detected with SARS-CoV-2 [36]. Later in August, it was reported that the wastewater from a catchment area in Australia was detected with positive results within 6 days [37]. All these facts suggest that the virus can be transmitted by the contaminated food package and water [38]. Therefore, risk-management approaches should be adopted to inspect the potential infected food, especially the cold chain food.

Food products are often traded in supermarkets, chain stores, and wholesale markets with large crowds, enclosed rooms, and high visitor flowrate, which makes it easy for the virus to be transmitted through droplets or aerosols. When there is one employee infected with SARS-CoV-2, it would be a disaster for all the people visiting there. That is why some of the beef packing and meat processing companies in the United States announced plant closures by the middle of April 2020 [39]. Studies have found that SARS-CoV-2 can be transported via the surface of contaminated droplets in the form of aerosol, which appears in the air at the entrance of the department store [28]. Thus, at the end of the food chain, people still suffer from the risk of infection in food consumption.

For preventing the potential spreads of the virus in the food chain, laws and regulations related to the production and sales of foods should be evaluated. Relevant departments of the food enterprise should strengthen the training of employees on epidemic prevention knowledge, using the epidemic prevention equipment rationally to avoid personal and food infection. During transportation, the quarantine standards should be carried out and unnecessary links should be reduced to avoid infection risk. Reasonable use of information and communication technology can both facilitate quarantine and minimizes transportation delays. For instance, the constant monitoring practice for food docking and transport can contribute to the effective quarantine and disinfection. Combination of online and offline platforms for food sales is another way to prevent the spread of virus. In addition, food companies can implement the fixed-point delivery, thereby reducing unnecessary people contact. What is more, there is an urgent need to establish a complete food safety traceability system, one that works for the identification of potential risky foods and ensure the food safety. Moreover, preferred precaution measures such as good hygiene practices and cleaning and sanitization of kitchens’ and restaurants’ surfaces are also required. The prevention and control of the current COVID-19 epidemic requires not only strict prevention measures but also continuous innovation of the food safety traceability, as well as rapid and accurate analytical methods.

## 3. Sample Pretreatment in Different Detection Platforms for SARS-CoV-2

Prior to the detection of virus, the pretreatment for the food samples is an essential part [8]. Different detection platforms such as PCR or immunosensors involve methods for sample treatment that are different. In this section, different pretreatment technologies are summarized on the basis of different detection platforms such as nucleic acid assays, immunoassays, and others, aiming to provide a reference for SARS-CoV-2 detection in food samples.

### 3.1. Nucleic Acid Assays

The nucleic acid assay is an analytical method used for the detection of all kinds of pathogenic microorganisms by analyzing their DNA or RNA genetic sequence. Due to its simplicity, easy methodology, widely accepted standard operating procedures, and available reagents and equipment, polymerase chain reaction (PCR) is a preferred method, one that was firstly applied in nucleic acid amplification [40]. PCR is an enzymatic method that is usually used to amplify trace amounts of RNA sequence, one that has become routine and reliable techniques for the detection of food-borne viruses because of its high sensitivity and sequence specificity [41].

**Figure 3:**
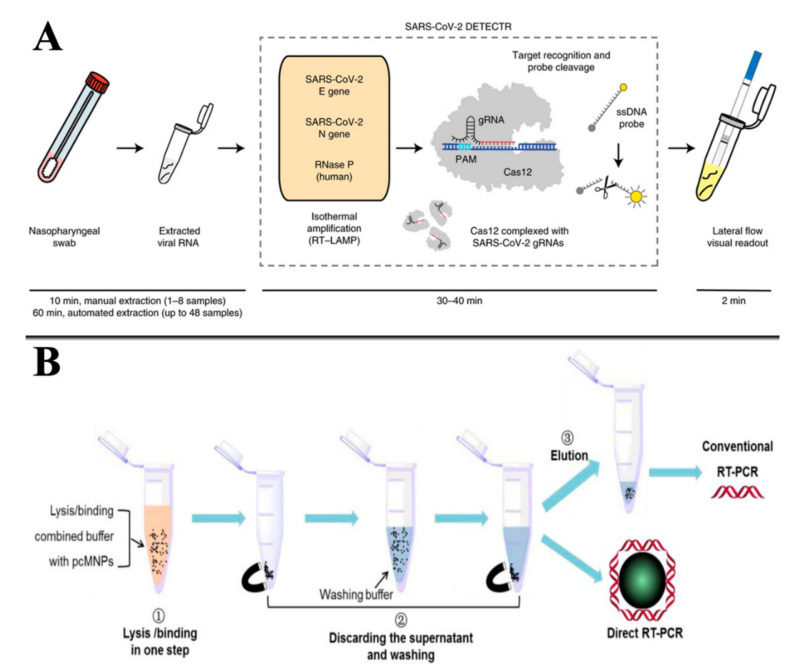
RNA extraction method for the detection of SARS-CoV-2: (**A**) conventional RNA extraction in Cas12-based detection; (**B**) magnetic nanoparticle-based viral RNA extraction in RT-PCR detection. Reproduced from [46,47] with permission from Elsevier and bioRxiv.

To meet the requirements of experiments and applications, researchers have continuously improved PCR-based techniques, and a variety of PCR techniques have been developed, such as fluorescent quantitative PCR, reverse transcription PCR, and reverse PCR [42]. On account of the good specificity and stability of DNA, other nucleic acid-based methods such as electrochemistry, fluorescence, and magnetic relaxing switching have been developed [43,44,45]. On the other hand, different methods are placed on the treatment of samples. For example, Broughton et al. reported a conventional RNA extraction method in Cas12-based detection of SARS-CoV-2, and Zhao et al. developed a simple magnetic nanoparticles-based viral RNA extraction method [46,47] (Figure 3). For food samples, we divided them into three categories: water, fruits and vegetables, and meat. In the following section, their pretreatment methods are discussed.

#### 3.1.1. Pretreatment of Water Samples

**Figure 4:**
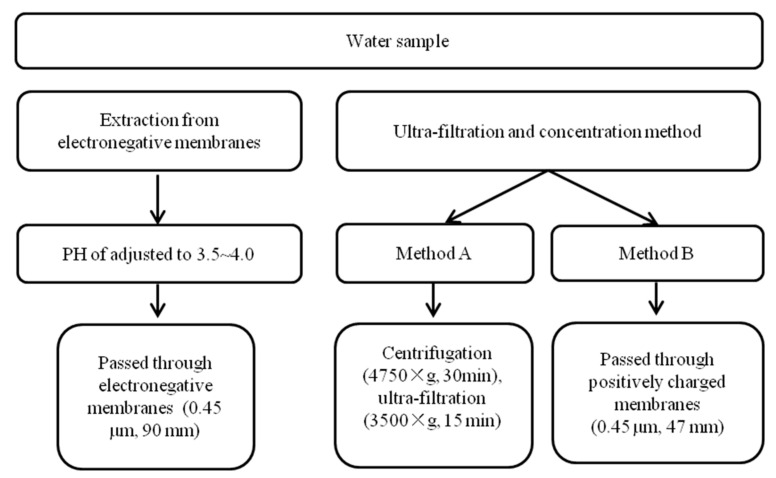
Summary of pre-treatment methods for nucleic acid determination of water samples [37,48].

As organic pollutants may exist in water samples, it is of great challenge to detect virus with low concentration. For the accurate detection, an appropriate pretreatment method is urgently needed to minimize interference from other components. Ahmed et al. reported two methods for the pretreatment of water samples: direct extraction method and ultra-filtration method [37,48] (Figure 4). The direct extraction method is achieved by electronegative membrane with adjusting pH, and RNA is directly extracted by using a kit after the water sample passes the negative membrane [37]. The other method, ultra-filtration and concentration, is conducted by using a kit to extract RNA, and then the sample is centrifuged, ultra-filtrated, and concentrated [48].

The extraction and ultra-filtration methods are successfully applied in the detection of SARS-COV-2 in wastewater samples by combining with reverse transcriptase quantitative polymerase chain reaction (RT-qPCR), confirming that they were applicable to the pretreatment of water samples for virus detection. Similarly, Hennechart-Collette adopted the ultra-filtration method for bottled water regarding the detection of norovirus (NoV) and hepatitis A virus (HAV) [49]. After the water samples were passed through a membrane (0.45 µm) followed by direct incubation at room temperature in a Petri dish with lysis buffer, RNA extraction procedure was performed and finally detected by reverse transcriptase quantitative polymerase chain reaction (RT-qPCR).

#### 3.1.2. Pretreatment of Fruits, Vegetables, and Their Products

Foods such as fruits, vegetables, and their products are susceptible to be contaminated by various bacteria, parasite eggs, or even viruses during the process of watering and fertilizing. However, due to the low abundance and uneven distribution of possible infected viruses in fruits, vegetables, and their products, a reasonable pretreatment method is required to achieve an accurate detection.

To evaluate the contamination of virus on foods, Yang et al. and Melgao et al. used known viruses to artificially infect fruits and vegetables [50,51]. In their work, direct treatment, elution and concentration, elution and flocculation techniques were applied to obtain the virus from food matrices, and then the RNA was extracted and detected by RT-PCR. Direct treatment was simply conducted by mixing food substrate with buffer, and then separated by centrifuging, wherein the supernatant was collected. The method is easy to operate but usually behaves with low enrichment efficiency, which has not widely been adopted. Hence, we mainly compared and analyzed the other two methods (elution and flocculation).

**Figure 5:**
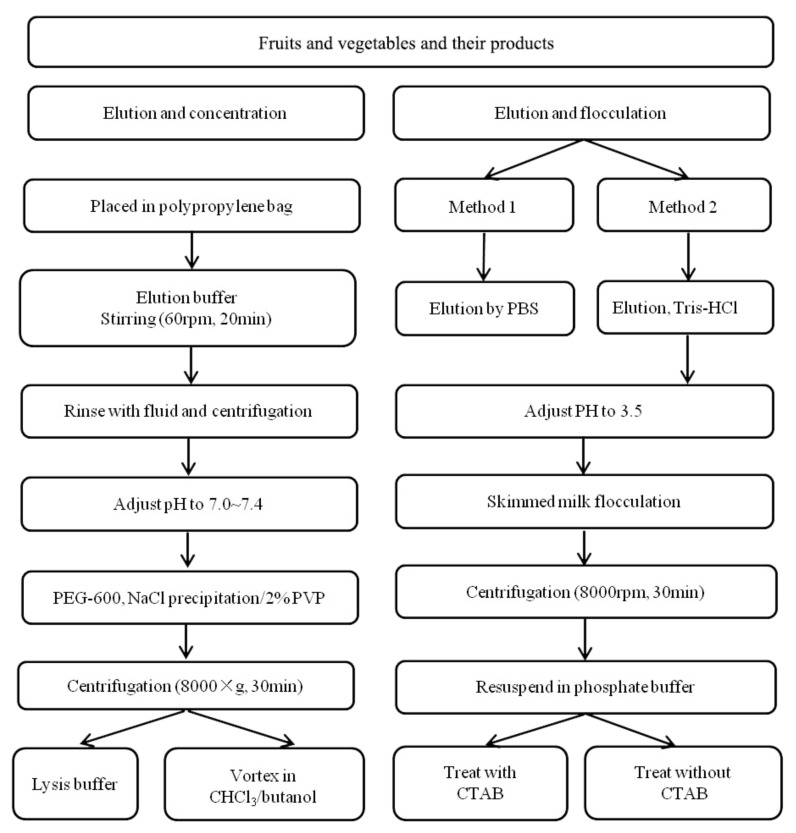
Summary of pretreatment methods for nucleic acid determination of fruits, vegetables, and their products [50,51].

The virus elution method is a way of eluting the virus particles off the surface of the food matrices with the appropriate buffer, and then further concentrating the elution solution by filtration and centrifugation. The pH of elution solution has a significant influence on the elution effect of virus particles. In most cases, an alkaline buffer of pH 9–10.5 is used to elute viruses on the surface of food because the number of virus particles attached to the food surface will be enhanced in an acidic environment. The procedures follow standard operations and can be applied to most of the food samples. For the flocculation method, it is carried out by coupling with elution, which includes two main steps: elution with alkaline buffer and lowering the pH to cause protein precipitation, followed by centrifugation and resuspension in a neutral buffer. The method requires no complicated equipment and only a small quantity of reagents, and thus it can achieve high efficiency and low cost. Both of the two methods can achieve satisfactory results for NoV detection in food samples [50,51]. The methods are compared in Figure 5, which suggests an alternative to pretreat fruits and vegetables for SARS-CoV-2 assays.

Moreover, Tahk et al. compared the three methods of polyethylene glycol precipitation, ultrafiltration membrane, and immunomagnetic separation for the concentration of HAV virus eluate in cabbage, lettuce, and sesame leaves [52]. For immunomagnetic separation, the surface of magnetic beads was first functionalized and modified with the antibody. After that, the magnetic beads were mixed with the sample eluent and incubated with rotation to release the enriched virus. Next, the magnetic beads were separated with a magnetic powder concentrator. Finally, the viral RNA extraction was conducted using a viral RNA mini kit and determined by RT-PCR. The magnetic separation treatment behaved with high efficiency and simple operations, which were able to achieve quick and efficient separation and avoid the complex interferences from food substrate.

#### 3.1.3. Pretreatment of Meat and Its Products

**Figure 6:**
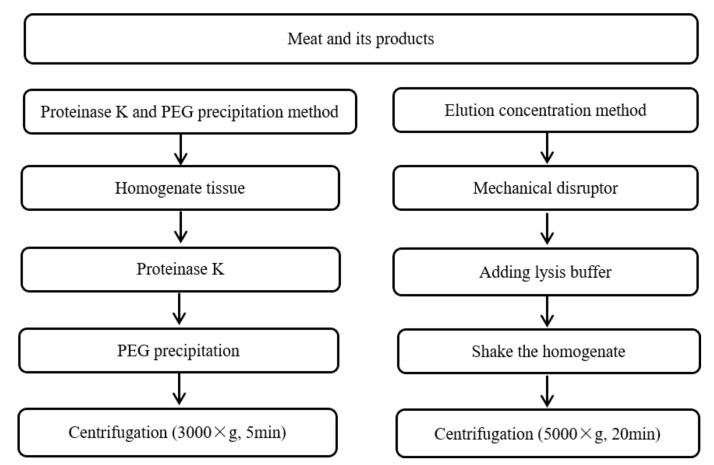
Summary of pre-treatment methods for nucleic acid determination of meat and its products [56,57,58].

Various studies have been reported for virus detection, for example, the analysis of avian influenza virus (H5N1), NoV, and hepatitis E virus (HEV) in meat and its products [53,54,55]. In using these methods to account for and summarize the pretreatment of foods, we can develop applicable methods for the detection of SARS-CoV-2 in meat and its products (Figure 6).

Zhang et al. employed the proteinase k-PEG precipitation method to extract and treat the tissue homogenates of oyster, with good enrichment effect for NoV detection [56]. Di Bartolo et al. and Markantonis et al. used an elution and concentration method to treat different meat products and detect HEV and NoV by RT-qPCR [57,58]. Kim and Oh described the elution and concentration method for the pretreatment of beef, followed by steps such as mechanical crushing, RNA splitting, oscillation homogenization, and centrifugation occurred, and finally RNA was extracted by the kit and detected via loop-mediated isothermal amplification lateral flow assay (LAMP-LFA) [59]. The elution and concentration method owns the advantages such as time saving, high efficiency, and low cost; however, this pretreatment method is easily disturbed by the external environment.

### 3.2. Immunoassays

Differing from the nucleic acid assays using the specificity of RNA or DNA, immunoassay uses the high selectivity of the antigen–antibody reaction to detect the virus, that is, the antigen can bind to the antibody specifically. The detection is carried out through the specific recognition reaction of the antigen and antibody, including enzyme-linked immunosorbent assay (ELISA), lateral flow assay (LFA), fluorescence immunoassay, and electrochemistry immunoassay, among others [60,61,62]. Immunoassay can be used for quantitative or qualitative analysis of a virus with the advantages of high sensitivity and specificity. To obtain stable signal-output and sensitive detection, specific antibodies, high-performance enzyme labels, and robust analytical technique are required, but an efficient pretreatment is fundamental for the immunoassay. As such, the food sample pretreatment, separation, and purification of antigen and antibody are very important. Two immunoassay strategies are usually included: virus detection and its antibody detection. On the basis of their differences, one can separate pretreatment for virus detection and pretreatment for antibody detection.

#### 3.2.1. Pretreatment Based on Virus Detection Methods

**Figure 7:**
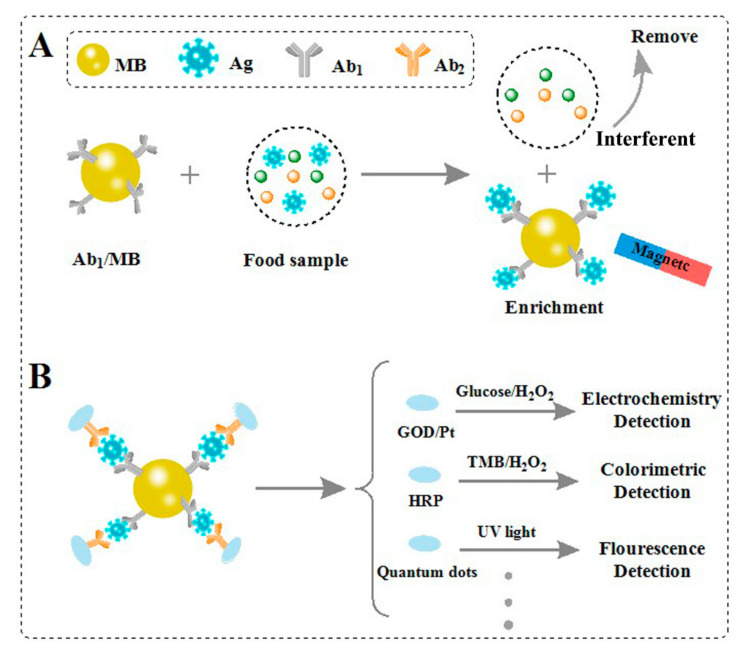
Schematic presentation of virus detection in food samples: (**A**) the magnetic separation technique for virus; (**B**) different immunoassays for virus [63,64].

The detection of a virus, also known as direct immunoassay, includes direct ELISA, sandwich ELISA, and competitive ELISA model, which needs low requirements and produces sensitive signal [63]. On the basis of the antigen–antibody recognition model, virus detection only requires the mixing of samples with designed probes. The commonly used pretreatment in immunoassays is based on the magnetic separation technique, as shown in Figure 7.

For example, Oh et al. proposed the magnetic separation for the separation and enrichment of influenza virus (H1N1) in practical samples [64]. Firstly, the Fe_3_O_4_ nanoparticles were modified with anti-influenza A virus antibody (MB-Ab1). Then, MB-Ab1 was used to specifically recognize H1N1 and form the MB–Ab1–H1N1 complex, and other moieties were able to be removed by washing with buffer. After that, the enzyme labelled Ab2 was able to be further hybridized with the complex, realizing the visual signal readout. This magnetic method possesses the advantages of simple separation, convenient enrichment, sensitive signal readout, and good anti-interference ability for the detection of influenza virus (H1N1).

Weerathunge et al. proposed a new nanozyme aptasensor strategy for the detection of the infective murine norovirus (MNV) without requiring specific concentration of the virus and removal of inhibitory compounds [65]. The strategy involves the noncovalent adsorption of the MNV-specific aptamer molecules on to the surface of gold nanoparticles (GNPs), leading to the loss of its inherent nanozyme activity. When incubating the sensor probe with MNV, the aptamers desorb from the surface of the GNPs and result in the recovery of the nanozyme activity, producing a blue color. Here, we name the method as “aptamer competitive model”, which offers an alternative for practical deployment of the norovirus detection with simple pretreatment in contaminated food.

Wu et al. proposed a digital single virus immunoassay for the simultaneous detection of H9N2, H1N1, and H7N9 avian influenza virus using fluorescent magnetic multifunctional nanospheres as both capture carriers and signal labels [66]. The magnetic property of nanospheres can realize efficient capture and separation of targets without sample pretreatment. Combining magnetic separation with digital single virus assay can achieve high sensitivity and selectivity in complex samples. However, the method suffers from some limitations—for example, it requires careful control to conjugate antibodies on the nanospheres, and the stability of probe is remained to be optimized. To overcome the limitation of antibody, one can utilize aptamers with high affinity and stability as a good alternative [67].

#### 3.2.2. Pretreatment Based on Antibody Detection Methods

Antibody detection is another method to reflect the virus level in immunoassay. It is known that a large number of antibodies can be produced in human or animal bodies as the antigen-like virus enters the body. According to the glycoprotein on the surface of the virus, the corresponding antibodies can be obtained to specifically recognize the virus, which is used for antibody immunoassay. For example, the antibody that reacts to the nucleocapsid glycoprotein (N) of a virus is widely applied to virus detection, in which the generated antibody can sensitively reflect the concentration level of the virus. As antibody produces in the living organisms and the samples are usually collected from blood, saliva, and urine, the pretreatment mainly includes ultra-filtration, centrifugation, and concentration.

Many pretreatments of antibody detection have been reported for the diagnosis of viruses. For example, Wu et al. developed a versatile immunosensor for the dilution detection of porcine circovirus type 2 (PCV2) antibody [61]. The positive serum sample was obtained from swine blood via coagulation and centrifugation. For the detection in serum substrate, the authors diluted newly obtained serum samples with phosphate-buffered saline (PBS) (0.1 M, pH 7.4), which was then directly used for the antibody detection. Similarly, Ragan et al. reported a fluorescence microsphere immunoassay for the detection of antibodies against Rift Valley fever virus (RVFV) glycoprotein (Gn) and nucleocapsid protein (Np) [68]. In the assay design, all the sera were inactivated by adding Tween 20 (0.25%, 1:10) and heating samples to 60 °C in a water bath for 2 h. Then, antibody response was detected by indirect ELISA using recombinant RVFV Gn and Np as antigens. The sample pretreatments were introduced to provide new ideas for food samples treatment, especially the liquid food samples.

#### 3.2.3. Pretreatment Based on Other Detection Methods

In addition to the magnetic separation and aptamer competitive strategy mentioned in virus detection, section staining observation can be used in immunohistochemistry analysis. For example, Ga and Sizhu reported an immunohistochemistry analysis of hepatitis E virus (HEV) in Tibetan pig liver tissue [69]. For the tissue samples treatment, they were cut into small pieces, fixed in neutral buffered formalin (10%), and embedded in paraffin. For immunohistochemistry, the tissues were fetched out, hydrated, and water bath-heated for antigen retrieval. After that, the sections were incubated with anti-HEV monoclonal antibody, followed by washing them with PBS and incubating with horse radish peroxidase (HRP)-labelled secondary antibody. Finally, 3,3-diaminobenzidine as chromogen was added to stain the section for observation under a microscope. The method presented standard operations for the detection of HEV with solid samples, which provided a new idea for the treatment of solid food samples.

Generally, nucleic acid assay requires pretreatments such as direct extraction and ultra-filtration for water samples; for fruits and vegetables, techniques such as elution and concentration, and elution and flocculation are adopted; for meat and its products, direct extract, elution, and concentration methods are applied to deal with them. Moreover, polyethylene glycol precipitation, ultrafiltration membrane, and magnetic separation have been used for treating food samples. Besides the above methods, serum sample and aptamer competitive model are widely used in immunoassay. All the pretreatment methods provide basic conditions for the detection of viruses. After introducing the pretreatment of food samples, we summarized the detection methods of SARS-CoV-2 in foods.

## 4. Advances in Methods for Detection of SARS-CoV-2 in Foods

Virus isolation and identification is one of the traditional methods for diagnosing epidemic virus infections, but virus culture usually requires several consecutive days, and the sensitivity and specificity are lower than nucleic acid detection. Although this method plays a key role in the identification of pathogens in the early stage, it is not suitable for the screening of large number of samples due to its time-consuming, cumbersome, and inefficient operations. This review does not cover the discussion on traditional methods. The current detection platforms for SARS-CoV-2 mainly include nucleic acid assays and immunoassays. In this section, design and application of these popular methods for detection of SARS-CoV-2 in food is reviewed and discussed.

**Figure 8:**
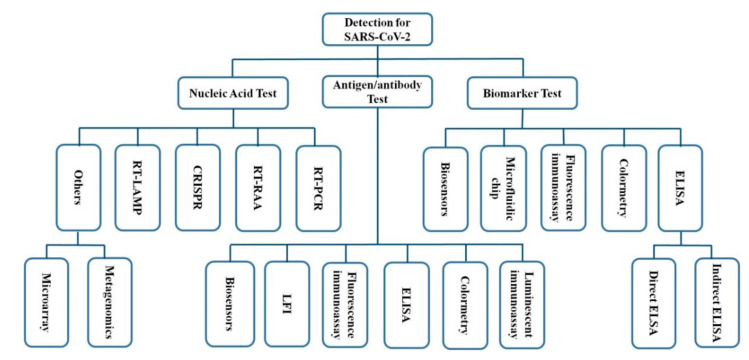
A summary of various methods that can be applied in SARS-CoV-2 detection (ELISA: enzyme-linked immunosorbent assay; LFI: lateral flow immunoassay [46,70,71,72,73,74,75,76,79,80,81,82,83,84,85,86]).

Nucleic acid assay coupled with PCR is the gold standard of virus detection, which owns high accuracy and specificity due to the stability of RNA. Since SARS-CoV-2 is highly contagious and pathogenic to humans, the direct immunoassay for the virus is not approved. However, the antibody detection can be conducted by sampling blood. Antibody immunoassay is a simple, sensitive, and rapid detection method in clinical diagnosis, one that can be used as a supplement of nucleic acid assay to screen COVID-19 cases. Additionally, other methods such as colorimetric methods and biomarker detection are potential alternatives to construct combined methods with nucleic acid assay for the detection of virus, including SARS-CoV-2 (Figure 8).

### 4.1. Nucleic Acid Detection

Nucleic acid detection is one of the most commonly used methods in SARS-CoV-2 detection, mainly including reverse transcription polymerase chain reaction (RT-PCR), clustered regularly interspaced short palindromic repeats (CRISPR) system, reverse transcription recombinase-aided amplification assay (RT-RAA), reverse transcription loop-mediated isothermal amplification (RT-LAMP), metagenomics method, and so on. The specific RNA sequence of SARS-CoV-2 can be used as an indicator to reflect COVID-19 disease [70]. Similarly, in the process of food processing, storage, and transportation, it may also be contaminated by the novel coronavirus. Prior to the qualitative or quantitative detection of SARS-CoV-2, certain measures can be adopted to treat food or food packaging.

Roche Diagnostics has developed a fully automated cabas 6800/8800 system on the basis of real-time RT-PCR technology for the qualitative detection of SARS-CoV-2. The test is a single-well dual-target assay, including the specific detection of SARS-CoV-2 and its subgenus. The systems provide up to 96 results and a total of 1440 results for the cobas 6800 System and 4128 results for the cobas 8800 System, which behaves with high efficiency, and rapid and sensitive detection [71]. Moreover, the conserved region of the two independent SARS-CoV-2 virus genes, ORF1ab gene and N gene, are used to design specific primers and probes that can achieve double positive signals by a real-time fluorescent RT-PCR analysis [72]. By applying dUTP/uracil DNA glycosylase (dUTP/UDG) protocol, the detection accuracy can be further improved. However, to perform RT-PCR tests, it requires laboratory-level facilities with reliable supply of electricity, expensive instruments, and trained personnel.

The Daejeon Institute of Chemical Technology and the Korea Food Research Institute in Korea jointly developed and evaluated the RT-LAMP detection method to detect the genomic RNA of SARS-CoV-2 [73]. The authors designed and optimized two groups of RT-LAMP reactions, which showed a limit of detection as low as 100 copies of SARS-CoV-2 RNA. Further, the leuco crystal violet method was adapted for the RT-LAMP assay to achieve a colorimetric detection with higher throughput. The method can be utilized for sampling inspection of food packaging or imported products. The combination with colorimetric method can not only enhance the detection throughput, but also greatly reduce the operating time and steps.

On the basis of the Cas12b-mediated DNA detection platform, Guo et al. established a CRISPR-assisted protocol for SARS-CoV-2 detection, which showed a detection limit of 1 × 10^4^ copies mL^−1^ for SARS-CoV-2 pseudovirus, with no cross-reactivity observed [74]. Moreover, Broughton et al. developed a rapid, easy-to-implement, and accurate CRISPR-Cas12-based LFA for SARS-CoV-2 from respiratory swab RNA extracts [46]. The method provided a visual and faster alternative to the SARS-CoV-2 real-time RT-PCR assay. Moreover, Xue et al. reported two RT-RAA methods for the S gene and ORF1ab gene detection of SARS-CoV-2, which showed the sensitivity of 10 copies for the S and one copy for the orf1ab gene per reaction [75]. Clearly, all the nucleic acid assays are sensitive and accurate. However, the methods are limited by advanced equipment and professional operations.

Metagenomics is a sensitive pan-pathogen assay for infectious disease diagnosis and the discovery of novel pathogens, which is applied in the rapid and simultaneous detection of SARS-CoV-2, the cause of COVID-19, and co-infections in people with COVID-19 [76]. The application of metagenomics for SARS-CoV-2 and respiratory diagnosis is becoming highly relevant due to the global transmission of SARS-CoV-2. Since nucleic acid detection requires complex sample pretreatment, a professional operator, and a long-term period, other methods are usually combined to produce the test report. For the food samples, appropriate pretreatment methods should be chosen to deal with the effect of complex matrices.

### 4.2. Antibody Detection

Labeled immunoassay is one of the most important analytical methods in antibody detection, in which produced antibody can specifically bind to antigen and the labeled secondary antibody as signal output [77,78]. After the body is invaded by virus, a specific antibody will be produced, which can indicate the existence of a virus. On the basis of this, medical professionals widely apply antibody detection in the clinical diagnosis of a virus; however, no report of the detection has been proposed in foods or packaging as antibody cannot be produced in those items. Therefore, the antibody detection was introduced here only to support the researchers that aspire to develop new applications in SARS-CoV-2 detection.

Rapid detection methods, such as flow immunoassay measurement and enzyme-linked immunosorbent assay (ELISA), can be a reference for the monitoring of the COVID-19 epidemic. With the in-depth research of SARS-CoV-2, lateral flow immunoassay is a favorable method for the diagnosis of the virus due to its convenient operations, short detection time, and free use of no large-scale instruments and equipment. In the traditional strategy, lateral flow immunoassay has been carried out by detecting the single antibody using the colloidal gold nanoparticles (GNPs). Li et al. developed an immunoassay that can both detect immunoglobulin M (IgM) and IgG antibodies against SARS-CoV-2 virus within 15 min [79]. Chen et al. proposed a lateral flow immunoassay (LFI) method that uses lanthanide-doped polystyrene nanoparticles as a fluorescent reporter to detect anti-SARV-CoV-2 IgG in human serum [80]. The detection process requires 10 min, and the test results agreed well with those of RT-PCR, which revealed its great potential in monitoring COVID-19 and evaluating patients’ response to treatment.

Moreover, ELISA, a golden standard in immunoassay, is also applied in the detection of SARS-CoV-2. Roy et al. reported a quantitative ELISA for high-throughput sample analysis, with qualified accuracy, sensitivity, and specificity in quantitative measures of exposure and immunity [81]. The test offers a good opportunity to quantitatively define both exposure and levels of immunity to SARS-CoV-2. Furthermore, Tré-Hardy et al. studied the analytical and clinical performance of two ELISA tests, the NovaLisa SARS-CoV-2 and Platelia SARS-CoV-2 methods, detecting antibodies directed against the virus nucleocapsid protein [82]. The clinical sensitivity, specificity, kinetics appearance, and reproducibility are excellent for IgG, IgA, and total antibodies, especially if the cut-off is optimized, which further revealed that ELISA test is a reliable method for SARS-CoV-2 detection in combination with RT-PCR.

Here, we provide a reference for future SARS-CoV-2 research in foods, since it has been reported that SARS-CoV-2 has been found on the surface of supermarket shelves and food packaging. Both RT-qPCR and ELISA are commonly used for the detection of viruses but with different principles. Nevertheless, the RT-qPCR analysis outcompetes ELISA in detection robustness and sensitivity. The ELISA can balance the difficulties of RT-qPCR in intensive labor, false negative results, and past infection.

### 4.3. Virus Protein Detection

Besides the detection methods mentioned above, researchers have pursued new detection methods for SARS-CoV-2 in light of the disadvantages of the existed methods. For example, Seo et al. designed a field-effect transistor biosensor for the detection of SARS-CoV-2 by coating graphene sheets with a specific antibody against SARS-CoV-2 spike protein [83]. The spike protein can be detected at an ultralow concentration of 1 fg mL^−1^ in PBS and 100 fg mL^−1^ in clinical transport medium. The limit of detections (LODs) of 1.6 × 10^1^ plaque-forming units mL^−1^ (pfu mL^−1^) and 2.42 × 10^2^ copies mL^−1^ can be achieved in culture medium and clinical samples, respectively. The work proposed a highly sensitive assay in COVID-19 without sample pretreatment or labeling. Baek et al. developed an RT-LAMP assay for the detection of SARS-CoV-2 by targeting the nucleocapsid gene of the viral RNA with the primer sets [84]. The method displayed a LOD of 10^2^ RNA copies close to that of qRT-PCR and rapid detection span of 30 min combined with the colorimetric visualization. Moreover, taking advantages of targeted amplification and long-read, real-time nanopore sequencing together, Wang et al. developed a nanopore-targeted sequencing (NTS) for SARS-CoV-2 detection with a LOD of 10 standard plasmid copies per reaction [85]. Although the method required a relatively long analysis time of 6–10 h, it can simultaneously detect other respiratory viruses, observe mutated nucleic acid sequences, classify the SARS-CoV-2 types, and conduct NTS for clinical diagnostic tests, which is considered acceptable for clinical use.

In light of food safety, there is an increasing need for the real-time detection techniques monitoring different aspects of the food. The immunoassays such as LIA and ELISA can meet the demands well in terms of their low cost and short detection time; however, they suffer from the limitations of relatively low stability and sensitivity. Therefore, many strategies have been reported to enhance the performance of the immunoassay. For instance, Wu et al. presented a nanozyme-linked immunosorbent assay for the sensitive detection of porcine circovirus type 2 (PCV-2) antibody with the naked eyes using nanozyme and HAuCl_4_/H_2_O_2_ coloring system [78]. With the aid of nanozyme and the new coloring system, the proposed method behaved two orders of magnitude higher in detection sensitivity compared to that of conventional ELISA. Moitra et al. developed a colorimetric method using GNPs capped with thiol-modified antisense oligonucleotides specific for SARS-CoV-2 N-gene to diagnose COVID-19 cases from the separated RNA samples [86]. The constructed method can realize a LOD of 0.18 ng µL^−1^ for SARS-CoV-2 RNA within 10 min. The developed visual naked-eye methods can greatly improve the detection performance without the need of any complex instrumental techniques.

The studies aim to construct the powerful techniques for improving the analytical performance, which is accompanied with the enhancements of analytical accuracy, precision, detection limits, and sample throughput, thereby expanding the practical range of food applications. Regarding specific analytical techniques applied to solve different problems in food analysis, one of the more active areas is the development of sample preparation techniques, in good agreement with the complex nature of foods. Owing to the development of new technologies, the new trends in analytical techniques and applications in the food safety can resolve the present pandemic crisis to a large extent. The recent increases in technology to detect and quantitatively assess the contaminants in foods should be more widely understood. The performance of different detection methods for SARS-CoV-2 is compared and listed in Table 1.

## 5. Conclusions

The COVID-19 pandemic is continuing to challenge our health and affect our lives in many ways. SARS-CoV-2 is highly contagious and causes high morbidity and mortality, and it also has been posing a great challenge to food supply chain and food industry. The food chain is a possible means for the transmission of SARS-CoV-2, and unfortunately limited attention is paid on the food chain to ensure food safety by monitoring the environments where food is produced, processed, stored, delivered, marketed, and so on. Monitoring the risks is essential to avoid the potential harm in the post-COVID-19 era, especially for the safety of food supply chain, which is full of uncertainties. In this review, possible transmission routes in the food chain were summarized. Due to the essential roles of pretreatment methods for the virus-contaminated food samples, sample pretreatment methods according to different detection platform such as nucleic acid assays, immunoassays, etc., were specifically discussed, aiming to provide informative guidance for effective detection of SARS-CoV-2 in foodstuffs. Advanced strategies for virus direction in foods play a critical role in food safety and verification due to their good analytical performance, convenient use, rapid and on-site detection, and simple operations. Herein, recent advances in detection method for SARS-CoV-2 were reviewed and discussed. Early warning, tracing, and detection should be united as a complete system in order to mitigate the COVID-19 outbreak, in which the detection for SARS-CoV-2 occupies a major position as it not only concerns screening of populations but also monitoring of possible contaminated sources. Authors should discuss the results and how they can be interpreted from the perspective of previous studies and of the working hypotheses. The findings and their implications should be discussed in the broadest context possible. Future research directions may also be highlighted.

## Figures and Tables

**Table 1:** Comparison of performance of different detection methods for SARS-CoV-2.

Detection Methods	Pretreatment	Assay Time	Accuracy	Advantages	Disadvantages	References
Nucleic acid assay	Elution and concentration; extraction/ultra-filtration; organic flocculation; protease K and PEG precipitation	≈1 to 6 h	>95%	High efficiency and sensitivity, easy to realize large-scale detection	Require professional technicians, high cost and time-consuming	[46,87]
Antibody detection	Elution and concentration; extraction/ultra-filtration	≈15 min	>75%	Simple pretreatment and detection procedures, high sensitivity	Long window period, difficulty in early diagnosis	[88,89]
Antigen detection	Elution and concentration; centrifugation; extraction/ultra-filtration;	≈30 min	28.6%–81.8%	Simple operations, high selectivity, easy to be constructed	Lack of stability and relatively low sensitivity	[90,91]
Biomarker detection	Elution and concentration; centrifugation; extraction/ultra-filtration	≈1 h	Not applicable	High sensitivity and selectivity, easy and little sampling	Difficult to obtain stable biomarkers, lack of standard	[92,93]

## References

[1] Zhu N., Zhang D., Wang W., Li X., Yang B., Song J., Zhao X., Huang B., Shi W., Lu R. (2020). A Novel Coronavirus from Patients with Pneumonia in China, 2019. N. Engl. J. Med..

[2] Feng Y., Marchal T., Sperry T., Yi H. (2020). Influence of wind and relative humidity on the social distancing effectiveness to prevent COVID-19 airborne transmission: A numerical study. J. Aerosol Sci..

[3] Rabaan A.A., Al-Ahmed S.H., Haque S., Sah R., Tiwari R., Malik Y.S., Rodriguez-Morales A.J. (2020). SARS-CoV-2, SARS-CoV, and MERS-CoV: A comparative overview. Infez. Med..

[4] Yan R., Zhang Y., Li Y., Xia L., Guo Y., Zhou Q. (2020). Structural basis for the recognition of SARS-CoV-2 by full-length human ACE2. Science.

[5] Su S., Wong G., Shi W., Liu J., Lai A.C., Zhou J., Liu W., Bi Y., Gao G.F. (2016). Epidemiology, Genetic Recombination, and Pathogenesis of Coronaviruses. Trends Microbiol..

[6] Yang P., Wang X. (2020). COVID-19: A new challenge for human beings. Cell. Mol. Immunol..

[7] Cucinotta D., Vanelli M. (2020). WHO Declares COVID-19 a Pandemic. Acta BioMed..

[8] Benzigar M.R., Bhattacharjee R., Baharfar M., Liu G. (2020). Current methods for diagnosis of human coronaviruses: Pros and cons. Anal. Bioanal. Chem..

[9] Rizou M., Galanakis I.M., Aldawoud T.M., Galanakis C.M. (2020). Safety of foods, food supply chain and environment within the COVID-19 pandemic. Trends Food Sci. Technol..

[10] World Health Organization COVID-19 and Food Safety: Guidance for Food Businesses: Interim Guidance. https://www.who.int/publications-detail/covid-19-and-food-safety-guidance-for-food-businesses.

[11] He D., Zhao S., Lin Q., Zhuang Z., Cao P., Wang M.H., Yang L. (2020). Re-Analysis of the Relative Transmissibility of Asymptomatic Cases among Close Contacts. SSRN Electron. J..

[12] Zhang N., Chen W., Chan P.T.J., Yen H.-L., Tang J.W.-T., Li Y. (2020). Close contact behavior in indoor environment and transmission of respiratory infection. Indoor Air.

[13] Li Y., Qian H., Hang J., Chen X., Hong L., Liang P., Li J., Xiao S., Wei J., Liu L. (2020). Evidence for probable aerosol transmission of SARS-CoV-2 in a poorly ventilated restaurant. medRxiv.

[14] Anderson E.L., Turnham P., Griffin J.R., Clarke C.C. (2020). Consideration of the Aerosol Transmission for COVID-19 and Public Health. Risk Anal..

[15] Galbadage T., Peterson B.M., Gunasekera R.S. (2020). Does COVID-19 Spread Through Droplets Alone?. Front. Public Heal..

[16] Wong D.H.T., Mak S.T., Yip N.K.F., Li K.K.W. (2020). Protective shields for ophthalmic equipment to minimise droplet transmission of COVID-19. Graefe’s Arch. Clin. Exp. Ophthalmol..

[17] Wong S.H., Lui R.N., Sung J.J. (2020). Covid-19 and the digestive system. J. Gastroenterol. Hepatol..

[18] Zhang Z., Zhang L., Wang Y. (2020). COVID-19 indirect contact transmission through the oral mucosa must not be ignored. J. Oral Pathol. Med..

[19] Wilson N.M., Norton A., Young F.P., Collins D.W. (2020). Airborne transmission of severe acute respiratory syndrome coronavirus-2 to healthcare workers: A narrative review. Anaesthesia.

[20] European Food Safety Authority [EFSA] (2020). Coronavirus: No Evidence That Food is a Source or Transmission Route.

[21] Olaimat A.N., Shahbaz H.M., Fatima N., Munir S., Holley R.A. (2020). Food Safety During and After the Era of COVID-19 Pandemic. Front. Microbiol..

[22] Carducci A., Federigi I., Liu D., Thompson J.R., Verani M. (2020). Making Waves: Coronavirus detection, presence and persistence in the water environment: State of the art and knowledge needs for public health. Water Res..

[23] Hayashi T., Aboko K., Mandan M., Yaegashi N., Konishi I. (2020). Molecular analysis of binding region of an ACE2 as a receptor for SARS-CoV-2 between humans and mammals. bioRxiv.

[24] Galanakis C.M. (2020). The Food Systems in the Era of the Coronavirus (COVID-19) Pandemic Crisis. Foods.

[25] Jiang F., Deng L., Zhang L., Cai Y., Cheung C.W., Xia Z. (2020). Review of the Clinical Characteristics of Coronavirus Disease 2019 (COVID-19). J. Gen. Intern. Med..

[26] Deng J., Jin Y., Liu Y., Sun J., Hao L., Bai J., Huang T., Lin D., Jin Y., Tian K. (2020). Serological survey of SARS-CoV-2 for experimental, domestic, companion and wild animals excludes intermediate hosts of 35 different species of animals. Transbound. Emerg. Dis..

[27] Zhang Q., Zhang H., Gao J., Huang K., Yang Y., Hui X., He X., Li C., Gong W., Zhang Y. (2020). A serological survey of SARS-CoV-2 in cat in Wuhan. Emerg. Microbes Infect..

[28] Liu Y., Ning Z., Chen Y., Guo M., Liu Y., Gali N.K., Sun L., Duan Y., Cai J., Westerdahl D. (2020). Aerodynamic analysis of SARS-CoV-2 in two Wuhan hospitals. Nature.

[29] Jalava K. (2020). First respiratory transmitted food borne outbreak?. Int. J. Hyg. Environ. Health.

[30] Ong S.W.X., Tan Y.K., Chia P.Y., Lee T.H., Ng O.T., Wong M.S.Y., Marimuthu K. (2020). Air, surface environmental, and personal protective equipment contamination by severe acute respiratory syndrome coronavirus 2 (SARS-CoV-2) from a symptomatic patient. JAMA.

[31] Hadei M., Hopke P.K., Jonidi A., Shahsavani A. (2020). A letter about the airborne transmission of SARS-CoV-2 based on the current evidence. Aerosol Air Qual. Res..

[32] Otter J.A., Donskey C., Yezli S., Douthwaite S., Goldenberg S., Weber D.J. (2016). Transmission of SARS and MERS corona-viruses and influenza virus in healthcare settings: The possible role of dry surface contamination. J. Hosp. Infect..

[33] Van Doremalen N., Bushmaker T., Morris D.H., Holbrook M.G., Gamble A., Williamson B.N., Lloyd-Smith J.O. (2020). Aerosol and surface stability of SARS-CoV-2 as compared with SARS-CoV-1. N. Engl. J. Med..

[34] Ling Y., Xu S.B., Lin Y.X., Tian D., Zhu Z.Q., Dai F.H., Wu F., Song Z.G., Huang W., Chen J. (2020). Persistence and clearance of viral RNA in 2019 novel coronavirus disease reha-bilitation patients. Chin. Med. J..

[35] Chin A.W., Poon L.L. (2020). Stability of SARS-CoV-2 in different environmental conditions–Authors’ reply. Lancet Microbe.

[36] General Administration of Customs of the People’s Republic of China (GAC, 2020) Press Conference of the Joint Prevention and Control Mechanism of Epidemic Import the State Council. http://fangtan.customs.gov.cn/tabid/1071/Default.aspx.

[37] Ahmed W., Angel N., Edson J., Bibby K., Bivins A., O’Brien J.W., Choi P.M., Kitajima M., Simpson S.L., Li J. (2020). First confirmed detec-tion of SARS-CoV-2 in untreated wastewater in Australia: A proof of concept for the wastewater surveillance of COVID-19 in the community. Sci. Total Environ..

[38] Gormley M., Aspray T.J., Kelly D.A. (2020). COVID-19: Mitigating transmission via wastewater plumbing systems. Lancet Glob. Heal..

[39] Laura R. Meat Processing Plants Are Closing due to covid-19 Outbreaks. Beef Shortfalls May Follow. https://www.washingtonpost.com/business/2020/04/16/meat-processing-plants-are-closing-due-covid-19-outbreaks-beef-shortfalls-may-follow/2020.

[40] Fakruddin M., Mannan K.S.B., Chowdhury A., Mazumdar R.M., Hossain M.N., Islam S., Chowdhury M.A. (2013). Nucleic acid amplification: Alternative methods of polymerase chain reaction. J. Pharm. Bioallied Sci..

[41] Stals A., van Coillie E., Uyttendaele M. (2013). Viral genes everywhere: Public health implications of PCR-based testing of foods. Curr. Opin. Virol..

[42] Srivastava N., Kapoor R., Kumar R., Kumar S., Saritha R.K., Kumar S., Baranwal V.K. (2019). Rapid diagnosis of cucumber mosaic virus in banana plants using a fluorescence-based real-time isothermal reverse transcription-recombinase polymerase amplification assay. J. Virol. Methods.

[43] Yue H., Zhou Y., Wang P., Wang X., Wang Z., Wang L., Fu Z. (2016). A facile label-free electrochemiluminescent biosensor for specific detection of Staphylococcus aureus utilizing the binding between immunoglobulin G and protein A. Talanta.

[44] Guo Y., Zhao C., Liu Y., Nie H., Guo X., Song X.-L., Xu K., Li J., Wang J. (2020). A novel fluorescence method for the rapid and effective detection of Listeria monocytogenes using aptamer-conjugated magnetic nanoparticles and aggregation-induced emission dots. Analyst.

[45] Qi X., Wang Z., Lu R., Liu J., Li Y., Chen Y. (2021). One-step and DNA amplification-free detection of Listeria monocytogenes in ham samples: Combining magnetic relaxation switching and DNA hybridization reaction. Food Chem..

[46] Broughton J.P., Deng X., Yu G., Fasching C.L., Servellita V., Singh J., Zorn K. (2020). CRISPR–Cas12-based detection of SARS-CoV-2. Nat. Biotechnol..

[47] Zhao Z., Cui H., Song W., Ru X., Zhou W., Yu X. (2020). A simple magnetic nanoparticles-based viral RNA extraction method for efficient detection of SARS-CoV-2. bioRxiv.

[48] Ikner L.A., Soto-Beltran M., Bright K.R. (2011). New Method Using a Positively Charged Microporous Filter and Ultrafiltration for Concentration of Viruses from Tap Water. Appl. Environ. Microbiol..

[49] Hennechart-Collette C., Martin-Latil S., Guillier L., Perelle S. (2015). Determination of which virus to use as a process control when testing for the presence of hepatitis A virus and norovirus in food and water. Int. J. Food Microbiol..

[50] Yang Z., Mammel M., Papafragkou E., Hida K., Elkins C.A., Kulka M. (2017). Application of next generation sequencing to-ward sensitive detection of enteric viruses isolated from celery samples as an example of produce. Int. J. Food Microbiol..

[51] Gil-Melgaço F., Victoria M., Corrêa A.A., Ganime A.C., Malta F.C., Brandão M.L.L., Medeiros V.D.M., Rosas C.D.O., Bricio S.M.L., Miagostovich M.P. (2016). Virus recovering from strawberries: Evaluation of a skimmed milk organic flocculation method for assessment of microbiological contamination. Int. J. Food Microbiol..

[52] Tahk H., Lee K.B., Lee M.H., Seo D.J., Cheon D.-S., Choi C. (2012). Development of reverse transcriptase polymerase chain reaction enzyme-linked immunosorbent assay for the detection of hepatitis A virus in vegetables. Food Control.

[53] Das A., Spackman E., Thomas C., Swayne D.E., Suarez D.L. (2008). Detection of H5N1 High-Pathogenicity Avian Influenza Virus in Meat and Tracheal Samples from Experimentally Infected Chickens. Avian Dis..

[54] Rutjes S.A., Lodder-Verschoor F., van der Poel W.H., van Duijnhoven Y.T., de Roda Husman A.M. (2006). Detection of no-roviruses in foods: A study on virus extraction procedures in foods implicated in outbreaks of human gastroenteritis. J. Food Prot..

[55] Althof N., Trojnar E., Böhm T., Burkhardt S., Carl A., Contzen M., Kilwinski J., Mergemeier S., Moor D., Mäde D. (2019). Interlaboratory Validation of a Method for Hepatitis E Virus RNA Detection in Meat and Meat Products. Food Environ. Virol..

[56] Zhang L., Xue L., Gao J., Cai W., Jiang Y., Zuo Y., Liao Y., Qin Z., Wu H., Cheng T. (2020). Development of a high-efficient concentrated pretreatment method for noroviruses detection in independent oysters: An ex-tension of the ISO/TS 15216-2: 2013 standard method. Food Control.

[57] Di Bartolo I., Angeloni G., Ponterio E., Ostanello F., Ruggeri F.M. (2015). Detection of hepatitis E virus in pork liver sausages. Int. J. Food Microbiol..

[58] Markantonis N., Vasickova P., Kubankova M., Mikel P., Botsaris G. (2018). Detection of foodborne viruses in ready-to-eat meat products and meat processing plants. J. Food Saf..

[59] Kim J.-H., Oh S.-W. (2019). Development of a filtration-based LAMP–LFA method as sensitive and rapid detection of *E. coli* O157:H7. J. Food Sci. Technol..

[60] Wu F., Zhao S., Yu B., Chen Y.M., Wang W., Song Z.G., Yuan M.L. (2020). A new coronavirus associated with human respiratory disease in China. Nature.

[61] Wu L., Li X., Shao K., Ye S., Liu C., Zhang C., Han H. (2015). Enhanced immunoassay for porcine circovirus type 2 antibody using enzyme-loaded and quantum dots-embedded shell–core silica nanospheres based on enzyme-linked immunosorbent assay. Anal. Chim. Acta.

[62] Shao K., Zhang C., Ye S., Cai K., Wu L., Wang B., Zou C., Lu Z., Han H. (2017). Near-infrared electrochemiluminesence bio-sensor for high sensitive detection of porcine reproductive and respiratory syndrome virus based on cyclodextrin-grafted po-rous Au/PtAu nanotube. Sens. Actuators B Chem..

[63] Wu L., Li G., Xu X., Zhu L., Huang R., Chen X. (2019). Application of nano-ELISA in food analysis: Recent advances and challenges. TrAC Trends Anal. Chem..

[64] Oh S., Kim J., Tran V.T., Lee D.K., Ahmed S.R., Hong J.C., Lee J., Park E.Y., Lee J. (2018). Magnetic nanozyme-linked im-munosorbent assay for ultrasensitive influenza A virus detection. ACS Appl. Mater. Interfaces.

[65] Weerathunge P., Ramanathan R., Torok V.A., Hodgson K., Xu Y., Goodacre R., Behera B.K., Bansal V. (2019). Ultrasensitive Colorimetric Detection of Murine Norovirus Using NanoZyme Aptasensor. Anal. Chem..

[66] Wu Z., Zeng T., Guo W.J., Bai Y.Y., Pang D.W., Zhang Z.L. (2019). Digital single virus immunoassay for ultrasensitive mul-tiplex avian influenza virus detection based on fluorescent magnetic multifunctional nanospheres. ACS Appl. Mater. Interfaces.

[67] Cao C., Zhang F., Goldys E.M., Gao F., Liu G. (2018). Advances in structure-switching aptasensing towards real time detection of cytokines. TrAC Trends Anal. Chem..

[68] Ragan I.K., Davis A.S., McVey D.S., Richt J.A., Rowland R.R., Wilson W.C. (2018). Evaluation of Fluorescence Microsphere Immunoassay for Detection of Antibodies to Rift Valley Fever Virus Nucleocapsid Protein and Glycoproteins. J. Clin. Microbiol..

[69] Ga G., Sizhu S. (2020). Molecular Detection of Hepatitis E Virus in Tibetan Swine. Pak. J. Zool..

[70] Chen L., Liu W., Zhang Q., Xu K., Ye G., Wu W., Mei Y. (2020). RNA based mNGS approach identifies a novel human coro-navirus from two individual pneumonia cases in 2019 Wuhan outbreak. Emerg. Microbes Infect..

[71] Roche’s Cobas SARS-CoV-2 Test to Detect Novel Coronavirus Receives FDA Emergency Use Authorization and Is Available in Markets Accepting the CE Mark. https://www.roche.com/dam/jcr:fcdc2329-235a-4010-86cd-a328c3e1db0a/en/13032020-mr-cobas-sar-cov-2-euaandceiv-en.pdf.

[72] Kam K.-Q., Yung C.F., Cui L., Lin R.T.P., Mak T.M., Maiwald M., Li J., Chong C.Y., Nadua K., Tan N.W.H. (2020). A Well Infant with Coronavirus Disease 2019 with High Viral Load. Clin. Infect. Dis..

[73] Park G.-S., Ku K., Baek S.-H., Kim S.-J., Kim S.I., Kim B.-T., Maeng J.-S. (2020). Development of Reverse Transcription Loop-Mediated Isothermal Amplification Assays Targeting Severe Acute Respiratory Syndrome Coronavirus 2 (SARS-CoV-2). J. Mol. Diagn..

[74] Guo L., Sun X., Wang X., Liang C., Jiang H., Gao Q., Dai M., Qu B., Fang S., Mao Y. (2020). SARS-CoV-2 detection with CRISPR diagnostics. Cell Discov..

[75] Xue G., Li S., Zhang W., Du B., Cui J., Yan C., Huang L., Chen L., Zhao L., Sun Y. (2020). Reverse-Transcription Recombinase-Aided Amplification Assay for Rap-id Detection of the 2019 Novel Coronavirus (SARS-CoV-2). Anal. Chem..

[76] Van Tan L., Man D.N.H., Hang V.T.T., Khanh P.N.Q., Xuan T.C., Phong N.T., Yen L.M. (2020). SARS-CoV-2 and co-infections detection in nasopharyngeal throat swabs of COVID-19 patients by metagenomics. J. Infect..

[77] Wu L., Zhou M., Wang Y., Liu J. (2020). Nanozyme and aptamer- based immunosorbent assay for aflatoxin B1. J. Hazard. Mater..

[78] Wu L., Zhang M., Zhu L., Li J., Li Z., Xie W. (2020). Nanozyme-linked immunosorbent assay for porcine circovirus type 2 an-tibody using HAuCl4/H2O2 coloring system. Microchem. J..

[79] Li Z., Yi Y., Luo X., Xiong N., Liu Y., Li S., Zhang Y. (2020). Development and clinical application of a rapid IgM-IgG com-bined antibody test for SARS-CoV-2 infection diagnosis. J. Med. Virol..

[80] Chen Z., Zhang Z., Zhai X., Li Y., Lin L., Zhao H., Bian L., Li P., Yu L., Wu Y. (2020). Rapid and Sensitive Detection of anti-SARS-CoV-2 IgG, Using Lanthanide-Doped Nanoparticles-Based Lateral Flow Immunoassay. Anal. Chem..

[81] Roy V., Fischinger S., Atyeo C., Slein M., Loos C., Balazs A., Charles R. (2020). SARS-CoV-2-specific ELISA development. J. Immunol. Methods.

[82] Tré-Hardy M., Wilmet A., Beukinga I., Favresse J., Dogné J.M., Douxfils J., Blairon L. (2020). Analytical and clinical validation of an ELISA for specific SARS-CoV-2 IgG, IgA, and IgM antibodies. J. Med. Virol..

[83] Seo G., Lee G., Kim M.J., Baek S.H., Choi M., Ku K.B., Lee C.S., Jun S., Park D., Kim H.G. (2020). Rapid detection of COVID-19 causative virus (SARS-CoV-2) in human nasopharyngeal swab specimens using field-effect transistor-based biosensor. ACS Nano.

[84] Baek Y.H., Um J., Antigua K.J.C., Park J.H., Kim Y., Oh S., Kim Y., Choi W.S., Kim S.G., Jeong J.H. (2020). Development of a reverse transcrip-tion-loop-mediated isothermal amplification as a rapid early-detection method for novel SARS-CoV-2. Emerg. Microbes Infect..

[85] Wang M., Fu A., Hu B., Tong Y., Liu R., Liu Z., Shen G. (2020). Nanopore targeted sequencing for the accurate and compre-hensive detection of SARS-CoV-2 and other respiratory viruses. Small.

[86] Moitra P., Alafeef M., Dighe K., Frieman M., Pan D. (2020). Selective Naked-Eye Detection of SARS-CoV-2 Mediated by N Gene Targeted Antisense Oligonucleotide Capped Plasmonic Nanoparticles. ACS Nano.

[87] Becherer L., Borst N., Bakheit M., Frischmann S., Zengerle R., Von Stetten F. (2020). Loop-mediated isothermal amplification (LAMP)—review and classification of methods for sequence-specific detection. Anal. Methods.

[88] Petherick A. (2020). Developing antibody tests for SARS-CoV-2. Lancet.

[89] Long Q.X., Liu B.Z., Deng H.J., Wu G.C., Deng K., Chen Y.K., Huang A.L. (2020). Antibody responses to SARS-CoV-2 in patients with COVID-19. Nat. Med..

[90] Mak G.C., Cheng P.K., Lau S.S., Wong K.K., Lau C.S., Lam E.T., Tsang D.N. (2020). Evaluation of rapid antigen test for detection of SARS-CoV-2 virus. J. Clin. Virol..

[91] Lassaunière R., Frische A., Harboe Z.B., Nielsen A.C., Fomsgaard A., Krogfelt K.A., Jørgensen C.S. (2020). Evaluation of nine commercial SARS-CoV-2 immunoassays. MedRxiv.

[92] Garg M., Sharma A.L., Singh S. (2020). Advancement in biosensors for inflammatory biomarkers of SARS-CoV-2 during 2019–2020. Biosens. Bioelectron..

[93] Park H.H., Kim H.N., Kim H., Yoo Y., Shin H., Choi E.Y., Lee W. (2020). Acetylated K676 TGFBIp as a severity diagnostic blood biomarker for SARS-CoV-2 pneumonia. Sci. Adv..

